# Failures analysis of tube coating in Circulating Fluidized Bed (CFB) boiler

**DOI:** 10.1016/j.heliyon.2024.e26134

**Published:** 2024-02-09

**Authors:** Teguh Widjajanto, Djarot B. Darmadi, Yudy S. Irawan, Femiana Gapsari

**Affiliations:** Department of Mechanical Engineering, Faculty of Engineering, University of Brawijaya, MT Haryono 167, Malang, 65145, Indonesia

**Keywords:** Boiler, Circulating fluidized bed (CFB), Coating, Failure, Tube

## Abstract

Circulating Fluidized Bed (CFB) boiler often experience leaks in the wall tube due to corrosion and abrasion of the bed material after use that varies between 3 and 8 months. To avoid erosion corrosion, a coating was done in the form of Chrome Clad Tube Armor (CTA). In this research, a Failure Analysis (FA) was performed on the characterization of the boiler tube using several types of samples, which are Wall Tube Without Coating (WT) and CTA (in new condition and after eight months of use). Macro visual, Field emission scanning electron microscopes (FE-SEM), Fourier-transform infrared spectroscopy (FTIR), corrosion, and Thermogravimetric analysis (TGA) tests showed that the CTA type has better corrosion and thermal resistance. The hardness values of the CTA and WT coating substrates after eight months of use were 197.75 and 195.2 HV. The failure mechanism on the tube was caused by high temperatures (long-term overheating) and friction between the tube and the fluid or metal. Wall tubes in furnaces fail due to erosion and corrosion due to fluid and solid particle mixtures and environmental contact.

## Nomenclatures

ASME: American Society of Mechanical Engineers
CFB: Circulating Fluidized Bed
CE: Counter Electrode
CTA: Chrome Clad Tube Armor
EDS: Energy Dispersive X-ray spectroscopy
FE-SEM: Field Emission -Scanning Electron Microscope
FTIR: Fourier-Transform Infra-Red
RE: Reference Electrode
TGA: Thermogravimetric Analysis
WE: Working Electrode
WT: Wall Tube Without Coating

## 1 Introduction

The high level of abrasion of the bed material against the tube in the Circulating Fluidized Bed (CFB) boiler can cause leaks. Leaks occur in the wall tube of the CFB boiler, where the sand used as the bed in the boiler is heated to a temperature of ∼500 °C [1], then the airflow is added together with the input of coal. The ignition temperature of a Circulating Fluidized Bed (CFB) boiler is influenced by oxygen content and coal type, with a negative correlation with higher oxygen content, guiding startup burner design [2,3]. The high temperature of the sand causes the coal to burn immediately resulting in an increase of temperature [4]. Sand, coal particles, and limestone circulate in the boiler and the wall tube picks up the heat [1]. Deposits on the inner tube wall are causing a decrease in heat transfer efficiency and gradual erosion near leakage areas. Generally, coatings are used on equipment to prevent damage from environmental exposure [5,6]. Therefore, the use of coatings must be adjusted to environmental conditions, system workload, and other factors that determine the quality of the coating [[6], [7], [8]]. The selection of the right type of coating will increase the life of the tubes and is expected to increase the life of the equipment as planned [7].

During the coating process itself, there are still things that need attention. Most tube coating failures are caused by insufficient surface preparation [9,10], which then causes failure in the bonding of the coating to the substrate. Other common causes of failure are inappropriate temperature and thickness during coating, excessive moisture, and improper mixing of components [11,12]. Exhaust gas and ash particles are the cause of deposition on the surface of the tube, which reduces heat transfer and corrosion [[13], [14], [15]]. Coating wear is caused by a combination of delamination and bed particles that experience turbulence. The tube coating must be dense and thick because it must be able to form a protective oxide and delay the diffusion of corrosion ions into the tubes. The presence of carbide in the coating aims at increasing the hardness and density of the coating [16]. However, the results in the field show that coatings that do not contain carbide have a longer service life than the others. For this reason, an in-depth failure analysis is needed regarding the FA coating on tube boiler.

This research was conducted on WT and CTA tubes before and after experiencing a leak/failure. FTIR Spectroscopy was used to detect thermo-oxidatively degraded by-product molecules (such as carbonyl and amide groups). TGA was conducted to analyze oxidative and thermal stability and initial decomposition temperature. The combination of coating material characterization in this research was able to explain the effect of damage on coating degradation.

## 2 Methods

**Fig. 1:**
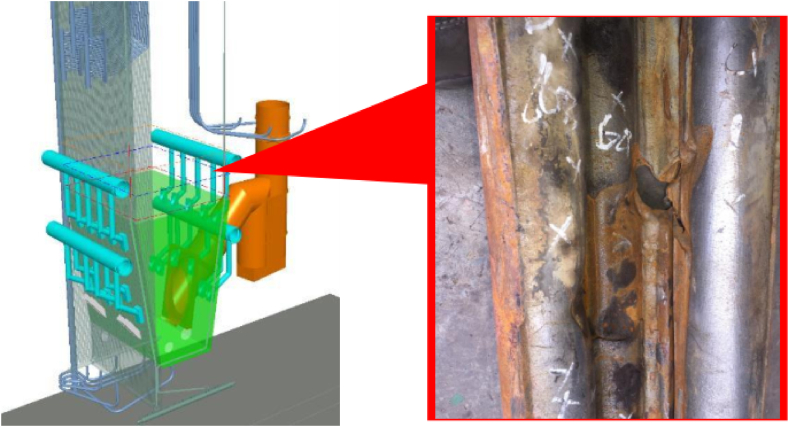
Sampling site.

This research utilized tubes. In a CFB boiler, the area that is prone to damage is the area where the fluidized bed circulates, which is the waterwall tube. The tube inner diameter was 46 mm with a diameter of 51 mm and a tube fin distance of 25.2 mm. The first sample was a water wall tube without coating (WT) with an ASME SA210 grade C substrate tube, with a chemical composition (%wt) of 98.67 Fe, 0.08 Cr, and 1.33 Mn. The thickness was 5 mm. The second sample is Chrom Clad Tube Armor (CTA) Coating. The CTA contained 38% Cr, 35% Ni, and 30% Fe. The hardness of the coating from the manufacturer was 465 HV and the hard phase was 1300HV. Location of sampling are shown in Fig. 1. Leakage of waterwall tube above refractory elevation 13,000 Leakage at 2 tubes corner area in the form of spot.

### 2.1 Boiler description

Amurang Steam Power plant used CFB Boiler with the capacity of 2 × 25 MW. For start-up unit, fuel oil can be used. When the temperature reaches 593 °C, the coal starts to be fed and the oil system will be deactivated when the temperature reaches 798 °C. The main steam temperature will meet the need of turbine steam turbine inlet parameter when the boiler load gets 60 % of the maximum continuous output. The total duration of the start unit should not exceed 7 h. When the rated load is achieved, the air excess was set until <20 % and the velocity of the flue gas on backpass heating surface area <12 m/s. The power plant boiler dimensions are 8 m (width) x 20.75 m (depth) x 37.585 m (height, steam drum centerline elevation) with boiler capacity of 38.97 m^3^ during normal operation. Coal will burn in the furnace, bed material (sand) will circulate with the coal to help distributing heat throughout the furnace. Unburnt coal will enter the cyclone through the loopseal and circulate back to the furnace. The fly ash from combustion will flow into the backpass along with the flue gas. Velocity or the ideal speed of the mixture of coal, air and bed material is 4 m/s with maximum speed of 4.5 m/s. By maintaining the speed, the flow in the furnace will be laminar and flow in the direction of the waterwall tube (does not cause abrasion on the waterwall wall). The volume of the combustion chamber of the furnace, cyclone etc. is about 1055 m^3^. The furnace cross section of the Amurang boiler power plant is 4876.8 m × 5791.2 m (28,242,524.16 m^2^), according to the manual book shown in Table 1. In addition to the furnace cross section, it is also stated that the furnace volume is 736 m^3^.

**Table 1:** Boiler parameters.

BOILER PARAMETERS	
MANUFACTURER	Shaanxi Northwest Power Generation Co., Ltd.
Rated evaporation capacity	120 t/h
SH steam pressure	9.8 MPa
SH steam temperature	540 °C
FW temperature	215 °C
Boiler efficiency	82.50%
Boiler type	CFB
Fuel type	Lignite
Furnace volume	736 m^3^
Furnace cross section area	28.2 m^2^
Furnace radiation heating surface	400 m^2^
Furnace volume thermal load	123.7 kW/m^3^
Furnace cross section thermal load	3225.9 kW/m^2^
Boiler inlet volume	106,800 Nm^3^/h
Boiler outlet volume	129,000 Nm^3^/h
Bed temperature	887 °C
Proportion between fly ash & bottom ash	9 : 1
Fuel heating value	4000 kcal/kg
Fuel consumption	21.96 t/h

The parameters in the power plant operation are detailed in Table 1.

**Fig. 2:**
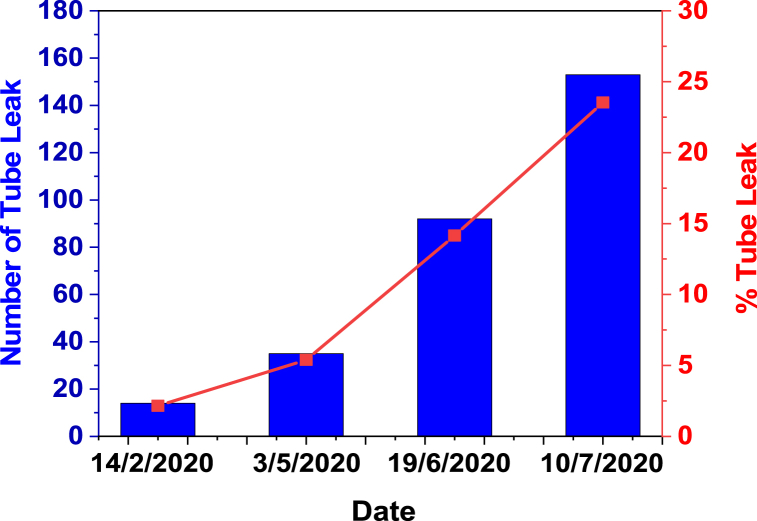
Boiler tube leak data.

Data on boiler tube leaks are shown in Fig. 2. Visual testing of specimens was carried out before (new) and after eight months of use, adjusted for the average leak time from WT.

### 2.2 Metallographic testing

Metallographic testing was carried out by taking photos of the macro and micro substrates, and the tube coating substrate cross-section was carried out using NIKON LDS R15 with 100X magnification. Testing was done in the form of a cross-section to determine the remaining thickness of the coating and substrate. On the substrate, the microstructure was etched with 5% nital.

### 2.3 Field emission scanning electron microscope (FE-SEM)

FE-SEM testing was performed with a Fe-SEM, JEOL JIB 4610. The analysis was also accompanied by an energy dispersive X-ray spectroscopy (EDS) detector.

### 2.4 Hardness testing

Hardness testing was carried out with Vicker microhardness equipment. WT and CTA hardness data were carried out after eight months of use. The results of the hardness test were in accordance with ASTM A370 standards.

### 2.5 FTIR (fourier-transform infrared) spectroscopy testing

Functional groups were analyzed by infrared absorption spectroscopy using FTIR testing. The wavelengths are between 4000 and 450 cm^−1^ with a scan rate of 10/minute using KBr powder matrix (Yang et al., 2017). Fourier-transform infrared spectrometer type 8400S/Shimadzu with a resolution of 4.0 cm^−1^ has been used to analyze the functional groups of coating tubes.

### 2.6 Thermogravimetric analysis (TGA)

The stability of the sample was like the change in mass at different temperatures of the tube coating specimen. Observations were made using the TGA instrument (TGA-1 Metler Toledo). The observation method referred to Maharana et al. (2020), where the powder specimen was weighed and then heated from ambient temperature to 500 °C (under a nitrogen atmosphere). The observation process was connected to computer software to obtain TGA data.

### 2.7 Corrosion testing

Corrosion testing was carried out with Autolab PGSTAT 204 N solution in 3.5% NaCl solution. The test employed the potentiodynamic polarization method. Corrosion testing was carried out with a 3-electrode system where Platinum served as the counter electrode (CE), Ag/AgCl (3 M KCl) as the reference electrode (RE), and tube coating as the working electrode (WE).

## 3 Results and discussion

### 3.1 Macro and micro analysis

**Fig. 3:**
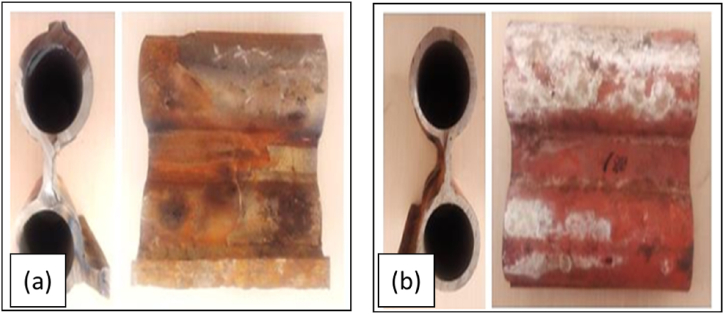
Visual samples of a) WT, b) CTA after eight months of use.

Visual observation of the surface of the water wall pipe without coating and with CTA coating is shown in Fig. 3.

Fig. 3a is the result of the WT macro visual inspection. The figure shows the surface of the pipe that had changed to a red-orange colour and with sediments on the entire surface of the pipe, which was the occurrence of a thorough corrosion process on the surface of the WT [17] and in the part where there was sediment [18]. The inner surface of the pipe appeared to have a duller appearance with deposits. These sediments might occur on the pipe wall during pipe processing and prior to coating application embedded within the coating. In addition, holes and cracks could be seen in the release of the sediment layer on the pipe surface due to surface corrosion attacks. This was different from the damage that occurred in heat exchanger pipe failures, which was the average erosion damage [18].

**Fig. 4:**
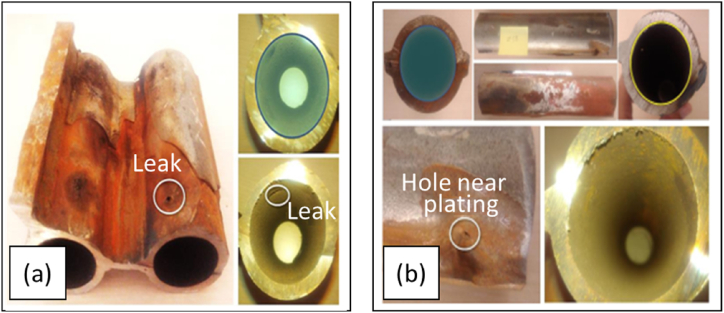
Surface macro photos of a) WT, b) CTA.

Different things were found on the surface of the tube with CTA (Fig. 3b). In Fig. 3b, it can be seen that the surface of the pipe was still good, corrosion did not occur throughout the surface, and holes due to corrosion occurred near the welds that were most likely the result of differences in the composition of the weld metal and the base metal. This also corresponds to Fig. 4. The existence of hole failure in the tube was generally caused by the presence of stress concentrations in different materials as a result of local heating so that the temperature distribution was uneven and in the end, a hole was formed in the tube [19].

After the visual analysis, macro photos were taken. Fig. 4 shows a macro photo of the surface.

**Fig. 5:**
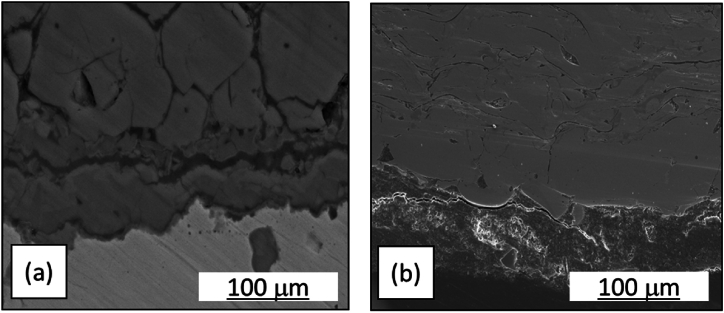
Micro photo of the cross-sections of a) WT, b) CTA.

**Fig. 6:**
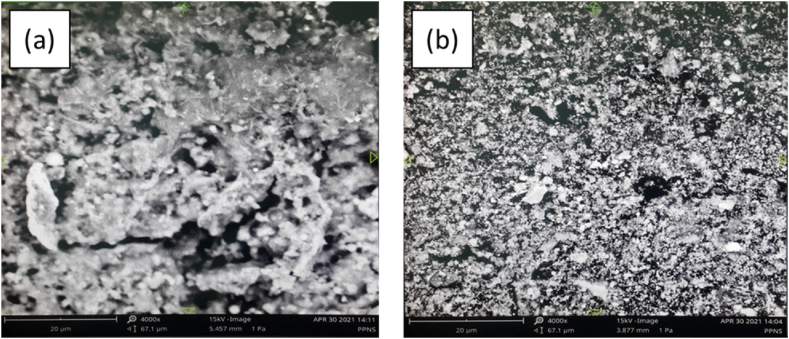
Micro photo of the substrates of a) WT, b) CTA.

Fig. 5 is a micro photo of the cross-sectional area for the WT and CTA samples. The micro photo of the WT sample Spreading cracks should be shown in Fig. 3, like pointing leak location in various directions on the corrosive surface (see Fig. 4a). This micro photo corresponded to the microstructure of the benchmark tube that had a microstructure of cracks in the transverse and rolling directions indicating a failure of the microstructure, compared to the coated benchmark tube. Some differences in damage were clearly visible [20]. The cracked and unstable passivation film (Fig. 5a) was insufficient to protect the surface from corrosion. Enlargement and interconnection of cavities resulted in cracks and chips being exfoliated from the seams [21]. On the other hand, in the CTA sample (Fig. 5b), it can be seen that the surface layer of the pipe cross-section was still intact, solid, and smooth. This surface indicated that there was no corrosive process or it was difficult to occur on the surface of the tube due to the isolation of the CTA coating. Pyrrhotite was also found as a fine platy crystal in the hollow of the tube [17]. The thickness of the corrosion products was usually about 0.04 mm, with corrosion products and sulfide crystals seen adhering to the surface (visual observation-Fig. 3). Furthermore, the temperature level received by the tube material during its application could be seen through its microstructure, such as changes in grain size, grain structure, spheroidization level, and so on, which could help determine the temperature level exposed to the material (see Fig. 6, micro photo of the substrates).

The micro photo of the substrate for sample 6a was a WT sample. The WT micro photo of the substrate looked larger and rougher with large surface cracks and voids. The large substrate micro photo was associated with the high corrosion rate of the WT tube. These results were in accordance with the test results on the micro photo of the cross-section in Fig. 5a, in which cracks and stringers were visible on the surface of the pipe and mimic microstructural damage. On the other hand, in Fig. 6b, the micro photo of the substrate had a smaller, denser, and uniform size, which was the result of a diffusion process on the CTA surface with the tube surface that in turn increases the hardness and corrosion resistance through attack by acid condensation and high pipe temperatures [22]. Overall, the substrate samples of WT and CTA micro photos were deposits that form iron carbide and iron oxide.

**Fig. 7:**
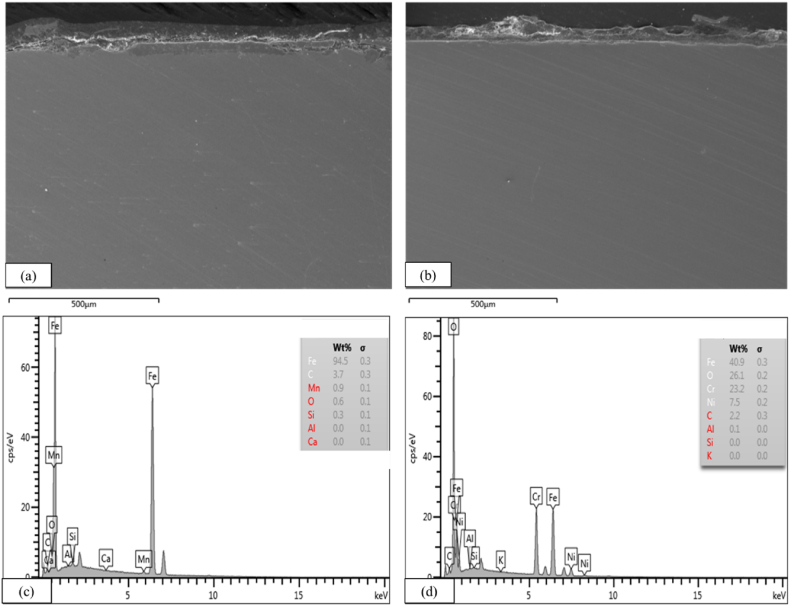
FE-SEM test photos of a) WT, b) CTA, EDS of c) WT, d) CTA.

The quantitative results of the surface composition were shown in Table 2. In terms of chemical composition, the EDS results in Table 2 shows that the contents of Fe were the main element in the formation of corrosion, where the value of the WT specimen had a higher percentage of Fe when compared to CTA, which was 94.5% compared to 40.9%. The high presence of oxygen in CTA indicates metal oxides formed in the form of a protective layer. The balanced transportation of metal and oxygen ions within an oxide can enhance the promotion of robust metal substrate adhesion, resulting in the formation of protective oxides [23]. The surface morphology of the WT and CTA specimens was seen using EDS mapping where the evaluation results showed that the effects of industrial corrosion appeared to be different. This was due to the degradation of the rough surface due to various corrosion mechanisms that occurred on the surface of the specimen [24]. On the other hand, in the WT specimen, the most prominent degradation occurred (see Fig. 7). The presence of Cr in CTA plays a crucial role in corrosion by altering the composition of the oxide layer formed during the hot corrosion process in an open system [25].

**Table 2:** Wavenumber of each of the WT and CTA samples.

Before Use (new)		Assignments	After 8 Months of Use		Assignments
WT	CTA		WT	CTA	
3411.15	3413.76	OH stretch	3435.55	3431.06	OH stretch
2924.74	–	CH2 stretch (asym.)		2927.04	CH2 stretch (asym)
–	2346.57	CH stretch	2346.54	2345.91	C=C conjugated
1639.14	1637.9	Arom. C=C stretch			
1454.31		CH aliphatic bending group	1731.84	1731.97	C–O stretch
1129.08	1135.5	C–O–C polysaccharide	1626.95	1627.27	Amide –I
645.98	646.74	CH out of plane aromatic band	1424.08	1440.78	Stretching -C-O inorganic carbonate
				1385.35	CH aliphatic bending group
				1266.14	C–N amide III band
			1165.23	1119.6	C–O–C polysaccharide
			1083.96	1018.96	C–O carbohydrate
				873	Bending –C=O inorganic carbonate
			694.94		CH out of plane aromatic band

### 3.2 Hardness analysis

**Fig. 8:**
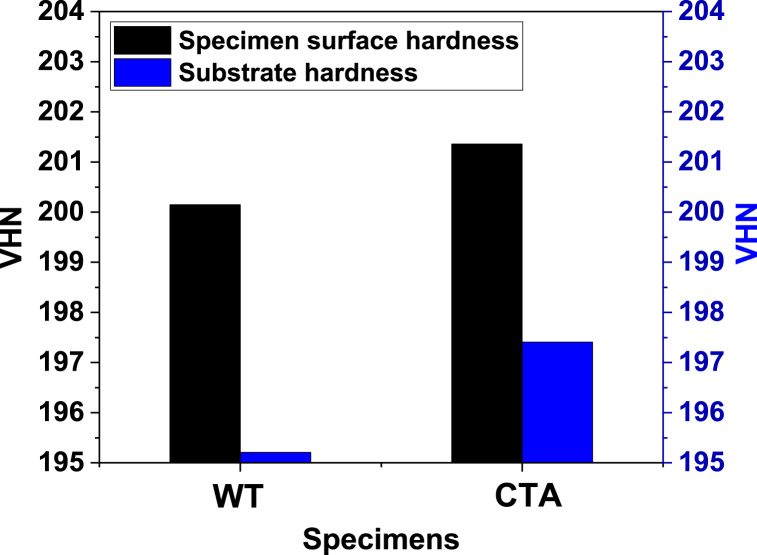
Hardness test results of WT and CTA.

The tube outer surface hardness value for each part of the sample (surface and substrate) is shown in Fig. 8. The surface hardness value for the tube surface with a coating of 201.5 HV then decreased by 0.65% (200.2 HV). This decrease was a result of the pipe surface corrosion process that involved some degree of degradation of the microstructure, which reacted with hydrogen through the cracks and microstructure stringers. This phenomenon could also be indicated by damage to the microstructure and could be related to the surface hardness data of the sample tube [26]. Furthermore, the hardness of the substrate was also shown in Fig. 8. From Fig. 8, it could be seen that the substrate hardness of the WT sample was around 195.2 HV. This substrate hardness value was lower than the substrate hardness value of the CTA tube, which was 197.75 HV. This increase in substrate hardness was a result of the diffusion phenomenon during the corrosion process. This result was in line with the micro photo of the substrates (see Fig. 3, Fig. 5) that had a smaller surface morphology.

### 3.3 FTIR analysis

**Fig. 9:**
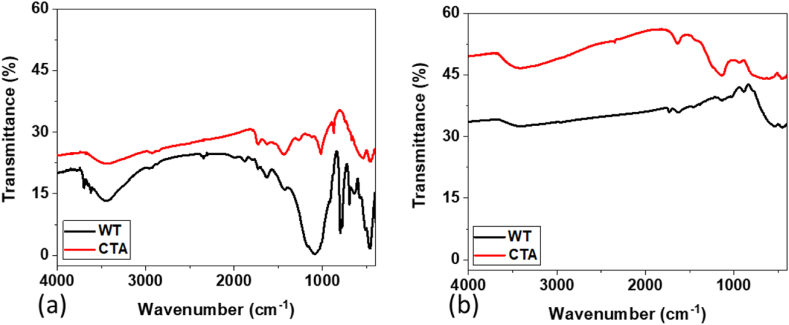
FTIR coating results (a) before and (b) after using the tube.

The FTIR spectra of the descaled layer of the WT and CTA samples before (new) and after eight months of use are shown in Fig. 9.

The results of the FTIR analysis showed the presence of wave absorption bands from the remaining coating that matched the CTA. It can be seen that apart from containing metal coatings, CTA also contained polymer coatings. This could be seen by the presence of polymer functional groups before use. More complete details were shown in Table 2. In general, there was an increase in the absorption of the sample wavenumber in the presence of CTA after eight months of use, so that the vibrational bands were compatible with CTA after use mixed with tube corrosion products, aliphatic-aromatic chains, and bending –C=O inorganic carbonate [[27], [28], [29]]. From Fig. 9a, it is known that there were about 5–6 absorption wave numbers with similarities in wave numbers 3411.15 cm^−1^ and 3413.76 cm^−1^, which were the first peaks that were OH strains and were usually associated with generic chemical-oxidative. Furthermore, the absorption bands in the WT and CTA samples before use appeared at 1639.14 (WT samples) and 1637.9 (CTA samples), which were related to carbonyl stretch [17]. In the sample after eight months of use, there was a stretch/increase in the absorption of the wave band to eleven peaks. The absorption peaks of the wave bands were very striking after eight months of use, starting from absorption at waves of 1731 cm^−1^ and 1627/1626 cm^−1^. The absorption wavenumbers at 1730 cm^−1^ and 1650 cm^−1^ could be indicated as the stretch of the carbonyl that could be related to the result of the generic thermo-oxidative degradation of the polymer after the use of the CTA coating [30]. The stretch on the wavenumber indicating the presence of a substrate from the CTA coating sample after eight months of use was found in 1626/1627 cm^−1^, 1424/1440 cm^−1^, 1165/1119 cm^−1^, and 873 cm^−1^ which respectively were Amide-I, Stretching -C-O inorganic carbonate, C–O–C polysaccharide, and Bending –C=O inorganic carbonate.

### 3.4 TGA analysis

**Fig. 10:**
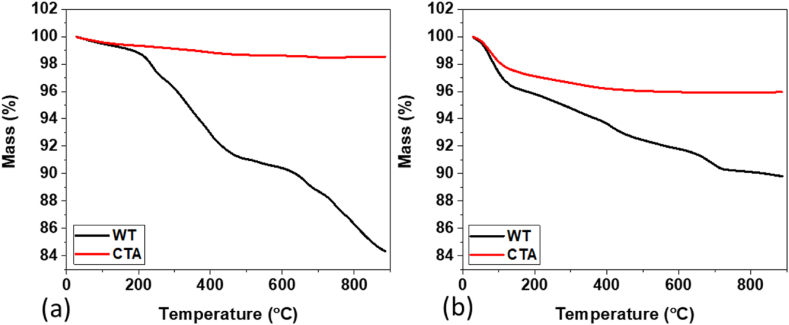
TGA WT and CTA curves (a) before (b) after eight months of use.

The thermal characteristics of the WT and CTA tube layers before and after use that were detached from the pipe wall are shown in the TGA curve in Fig. 10. From Fig. 10, it can be seen that the CTA samples had one decomposition stage both before and after eight months of use. The CTA sample before use hardly decomposed during the heating process from a temperature of 20–900 °C, due to the high thermal resistance of the CTA coating, which required a high temperature (+1000 °C) to vaporize the tube coating metal [31]. It was the same with the CTA samples after eight months of use. A glass transition occurred at a temperature of around 150 °C with a weight loss of 2%. These results are in line with the results of a study reported by Ernault et al. (2017) [31].

WT samples after eight months of use were seen to have higher thermal resistance compared to before use. The first stage of WT decomposition before use can occur at temperatures in the range of 20–200 °C that indicated the release of the bonds of water moisture and volatile organic compounds with a weight reduction of 2%. Meanwhile, in the WT samples after eight months of use, the first stage of decomposition occurred below a temperature of 200 °C with a weight loss of 3%. The second stage decomposition of WT before FA occurred at a temperature of 200–600 °C and the third stage occurred at 600–900 °C with the weight loss of WT samples of 10 and 17% for the second and third stages respectively. This sample decomposition process occurred through a thermo-oxidative process. Meanwhile, for WT samples after eight months of use, the second stage of decomposition took longer, which was from a temperature of 150–700 °C and the third stage of decomposition occurred from 700 to 900 °C with a smaller total weight loss of 10% with two exothermic features. From the percentage of sample residue after eight months of use, which was greater, it was known that the thermal resistance of the sample was also higher.

### 3.5 Corrosion analysis

**Fig. 11:**
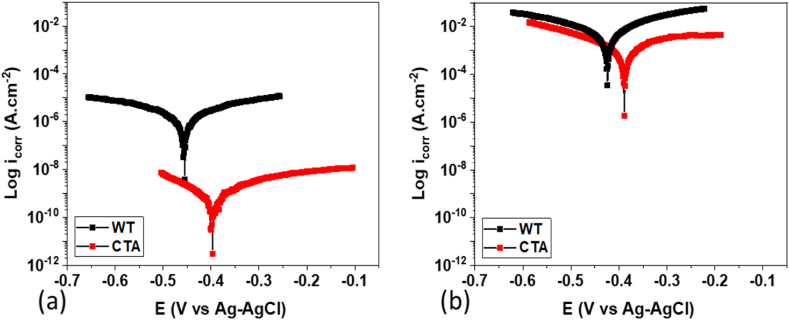
Corrosion test results (a) before and (b) after FA.

The potentiodynamic polarization curve types of the tubes (WT and CTA) before and after eight months of use for their active and passive corrosion regions are shown in Fig. 11 and the parameters related to corrosion are presented in Table 3. From Fig. 11a and b, it can be seen that both E_corr_ and i_corr_ CTA samples before and after eight months of use shifted to negative. This indicates that the CTA coating provided protection against tube corrosion [32] through termination of the anodic dissolution reduction reaction resulting in a decrease in i_corr_ value [33]. After investigation, after eight months of use there was a significant increase in the value of the corrosion rate from 0.0088 mm/year to 49.31 mm/year for the WT sample due to the corrosive conditions of the tube in the power plant. From Table 3, with a decrease in potential (E_corr_), the value of the passivation potential was also less. With the presence of a CTA coating, the chemical corrosion reactivity between the metal and its environment was also reduced and the reduction in the corrosion rate could be attained. The difference in corrosion rates obtained from the CTA samples before use was lower than the CTA samples after eight months of use.

**Table 3:** Corrosion rates of WT and CTA samples.

Variations	βa (V/dec)	-βc (V/dec)	E_corr_ (V)	i_corr_ (A)	Corrosion rate (mm/year)
WT before use	0.086	0.07	−0.46	8.45 × 10^−7^	0.009
CTA before use	0.12	0.28	−0.40	1.28 × 10^−9^	1.33 × 10^−5^
WT after eight months of use	0.14	0.10	−0.42	0.0047	49.31
CTA after eight months of use	0.28	0.32	−0.39	0.0026	27.06

### 3.6 Failure mechanism on tube coating

**Fig. 12:**
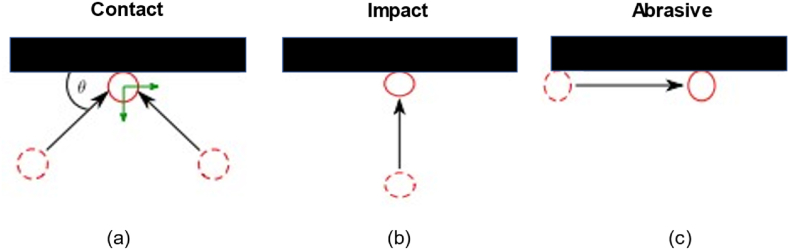
Contact angle causing wear (a) Contact with angle 0° > θ > 90°, (b) Contact with angle θ = 90°, and (c) Contact with angle θ = 0°.

The failure mechanism on the tube was caused by high temperature (long-term overheating) in the pipe. High temperatures made it easier for carbide to diffuse [15,16]. This caused changes in the microstructure of the boiler tube. Changes in the microstructure, hardness, and corrosion rate of the tube coating indicated other causes, namely that the failures that occurred were not only due to differences in carbide composition but also due to contamination with corrosive residues for a long time, and the location of the pipe damage was dominated by the oxygen element content [20]. In addition, there was also friction and adhesion between the pipe and the fluid. Particles of two materials that rubbed against each other caused wear on the surface, moreover, the alloy and hardness of the two rubbing materials were the same so they combined more easily to produce greater wear [33,34]. Impact and abrasion and shock were the causes of pipe wear failure due to friction with different angles. The surface of the WT pipe rubbed especially when dredging and transporting minerals. Therefore, the materials used had to be as hard as possible to resist abrasive wear, although their microstructure and surface were also important factors. The coating on the tube caused the surface of the tube to be hydrophobic thus reducing friction with the fluid. A sketch of the wear mechanism that caused tube failure was shown in Fig. 12.

The failure of wall tubes in furnaces can be attributed to erosion and corrosion resulting from the interaction between fluid and solid particle mixes, as well as exposure to the surrounding environment.

## 4 Conclusion

The characteristics of the tube with two types of samples, which were WT and CTA, were visually analyzed for failure using residue hardness, FTIR, and TGA. CTA had higher corrosion resistance because it was able to isolate the tube from the environment. Visual observation showed that the WT pipe was damaged (corroded all over the surface) more severely than the CTA tube. These results were in accordance with the results of surface strength and substrate hardness tests that had a higher hardness after being treated with the CTA. The coating component on the CTA pipe substrate was a polymer coating. FTIR spectra on WT and CTA also showed stretching of the FTIR functional groups due to diffusion. The CTA had higher hardness, corrosion rate, and thermal resistance than the WT. The hardness values of the CTA and WT coating substrates after eight months of use were 197.75 and 195.2 HV respectively. The corrosion rate after eight months of use at WT was 49.31 mm/year, while at CTA it was 27.06 mm/year. The failure mechanism on the tube was caused by high temperature (long-term overheating) on the tube. High temperatures caused carbide to diffuse more easily resulting in changes in the microstructure of the tubes. The coating on the tube caused the surface of the tube to be hydrophobic thus reducing friction with the fluid. Proper coating treatment of boiler tubes will increase the service life of the boiler. Further research is needed regarding coating compositions to increase the service life of CFB boiler.

## CRediT authorship contribution statement

**Teguh Widjajanto:** Writing – original draft. **Djarot B. Darmadi:** Supervision. **Yudy S. Irawan:** Supervision. **Femiana Gapsari:** Writing – review & editing, Validation, Supervision, Data curation, Conceptualization.

## Declaration of competing interest

The authors declare that they have no known competing financial interests or personal relationships that could have appeared to influence the work reported in this paper.
